# Situating space: using a discipline-focused lens to examine spatial thinking skills

**DOI:** 10.1186/s41235-020-00210-z

**Published:** 2020-04-22

**Authors:** Kinnari Atit, David H. Uttal, Mike Stieff

**Affiliations:** 1 Graduate School of Education, University of California, Riverside, 1207 Sproul Hall, Riverside, CA 92521 USA; 2 School of Education and Social Policy, Northwestern University, Evanston, IL USA; 3 Learning Sciences Research Institute, University of Illinois at Chicago, Chicago, IL USA

**Keywords:** Spatial skills, STEM education, Expertise, Interdisciplinary research, Discipline-based education research

## Abstract

Spatial skills are an important component of success in science, technology, engineering, and math (STEM) fields. A majority of what we know about spatial skills today is a result of more than 100 years of research focused on understanding and identifying the kinds of skills that make up this skill set. Over the last two decades, the field has recognized that, unlike the spatial skills measured by psychometric tests developed by psychology researchers, the spatial problems faced by STEM experts vary widely and are multifaceted. Thus, many psychological researchers have embraced an interdisciplinary approach to studying spatial thinking with the aim of understanding the nature of this skill set as it occurs within STEM disciplines. In a parallel effort, discipline-based education researchers specializing in STEM domains have focused much of their research on understanding how to bolster students’ skills in completing domain-specific spatial tasks. In this paper, we discuss four lessons learned from these two programs of research to enhance the field’s understanding of spatial thinking in STEM domains. We demonstrate each contribution by aligning findings from research on three distinct STEM disciplines: structural geology, surgery, and organic chemistry. Lastly, we discuss the potential implications of these contributions to STEM education.

## Significance statement

With more than 50% of science, technology, engineering, and math (STEM) majors leaving the STEM fields prior to graduation, there has been an increased focus on improving STEM retention rates at universities across the USA (National Science Foundation, 2018). Spatial skills are an important component of success in STEM fields and have been the focus of much psychological research for the last 100 years. Yet, only in the last few decades has the field of psychology come to recognize the complexity and nuance of the spatial tasks carried out by STEM experts during their practice. Consequently, a growing number of researchers are taking an interdisciplinary perspective on spatial thinking in STEM, with the aim of broadening our understanding of the kinds of spatial skills required to practice STEM domains. In a parallel effort, having a strong appreciation for the importance of spatial thinking to STEM reasoning and problem-solving, discipline-based education researchers have focused much research on understanding how to bolster students’ skills in completing domain-specific spatial tasks. This paper highlights critical lessons learned from interdisciplinary and discipline-based education research in three STEM fields—structural geology, surgery, and chemistry—and considers their implications for how to support students’ STEM learning and success at advanced educational levels.

## Introduction

There has been a strong national emphasis on increasing the number of individuals who pursue and succeed in STEM disciplines (Graham, Frederick, Byars-Winston, Hunter, & Handelsman, 2013; Hayden, Ouyang, Scinski, Olszewski, & Bielefeldt, 2011). An important component of success in these fields are spatial skills (Shea, Lubinski, & Benbow, 2001; Wai, Lubinski, & Benbow, 2009). Spatial skills enable us to manipulate, organize, reason about, and make sense of spatial relationships in real and imagined spaces. STEM professionals often employ spatial skills when completing tasks within their domain. For example, petroleum geologists use spatial skills when deciding on the location for a new oil well. They interpret and visualize the shapes and locations of three-dimensional (3D) geologic structures that exist under the ground from two-dimensional (2D) seismic data. The ubiquitous accounts of such practices among STEM experts have made spatial skills a focal point of research and a target of educational interventions.

Much of what we know about spatial skills today is a result of decades of psychological research conducted to understand the kinds of skills that make up this skill set (Shepard & Metzler, 1971). The psychometric tasks developed by researchers studying spatial thinking (Bennett, Seashore, & Wesman, 1947; Guay, 1977; Vandenberg & Kuse, 1978) measure isolated spatial skills independent of context, and have been suggested to provide the foundation for the field-specific spatial thinking required by STEM experts (Uttal & Cohen, 2012; Wai et al., 2009). In this paper, we will refer to these as *fundamental* spatial skills. More recently, the field of psychology has recognized that spatial problems in STEM domains vary widely, as they are situated within domain-specific contexts. For instance, the shapes of the geologic structures (e.g., faults or synclines) that occur in the environment dictate the kinds of spatial problems structural geologists need to solve. If the rocks that make up a geologic structure are bent, then the structural geologist has to visualize unbending them to understand the geologic history of the region (Atit, Shipley, & Tikoff, 2013). As a result, many psychological researchers have embraced a contextualized and domain-specific approach for examining these skills to gain a full understanding of the nature of spatial thinking in STEM domains and the role of spatial skills in the development of STEM expertise.

Corresponding to the interdisciplinary research conducted by psychologists to better understand the nature of spatial thinking in STEM, discipline-based education researchers have also pursued much research on spatial cognition in STEM disciplines. Discipline-based education research (DBER) “investigates learning and teaching in a discipline using a range of methods with deep grounding in the discipline’s priorities, worldview, knowledge, and practices” (National Research Council, 2012b, p. 9). Having a strong appreciation for the large number of spatial problems that STEM disciplinary experts face in their work, and recognizing that these kinds of problems are a source of difficulty for novices trying to learn the discipline (e.g., Alles & Riggs, 2011; Kali & Orion, 1996), many researchers conducting DBER have focused their efforts on identifying how to bolster students’ skills for completing spatial tasks within their respective domains (e.g., Harle & Towns, 2011; Reynolds et al., 2005; Sorby, 2007). This work provides a new, important perspective on the kinds of spatial problems that occur in STEM domains and on examining the nuances of how STEM experts and novices solve them.

In this paper, we will first briefly review the history of research on spatial thinking in psychology. Then, we will align more contemporary findings from interdisciplinary and DBER studies investigating spatial thinking in three distinct STEM disciplines—structural geology, surgery, and chemistry—and discuss four lessons emerging from this work for the field of spatial cognition. The contributions of the interdisciplinary research and DBER on spatial thinking include an increased understanding of the following: (1) spatial thinking in STEM domains is influenced by domain knowledge and is context-dependent, (2) the kinds of spatial problems measured on psychometric tests of spatial skills are more complex and multifaceted when they occur within STEM contexts, (3) existing psychometric measures do not capture all of the spatial skills required to solve STEM-specific spatial problems, and (4) STEM experts do not rely on their spatial skills uniformly to solve spatial problems they encounter during their practice. Lastly, we will discuss potential implications of these contributions and how they provide insight on how to bolster and develop STEM-relevant spatial skills in students.

## A brief history of the research on spatial thinking in psychology

Understanding spatial skills has been a topic of interest in psychology for much of the last hundred years (Bethell-Fox & Shepard, 1988; Carroll, 1993; Shepard & Metzler, 1971). Historically, the recognition of this class of cognitive skills has roots in the study of mechanical aptitude (Cox, 1928; Paterson, Elliot, Anderson, Toops, & Heidbreder, 1930) and in defining the factors of intelligence (Carroll, 1993; Thurstone & Thurstone, 1941). Once it was established that spatial skills were distinct from other sets of skills, such as verbal or mathematical skills, efforts by researchers shifted to identifying the different kinds of spatial skills (Linn & Petersen, 1985; McGee, 1979) and their underlying cognitive processes (Shepard & Metzler, 1971). Although the field generally agrees on the importance of fundamental spatial skills to spatial thinking (Shea et al., 2001; Wai et al., 2009) and that it comprises more than one skill (Linn & Petersen, 1985; McGee, 1979; Newcombe & Shipley, 2015), there is surprisingly little consensus about the details of what makes up this skill set (Caplan, MacPherson, & Tobin, 1985; Hegarty & Waller, 2004).

In trying to define the distinct kinds of fundamental spatial skills, hundreds of psychometric tests measuring isolated spatial skills, devoid of context, have been developed and employed. Most of these tests measure small-scale spatial skills that involve the visualization and manipulation of 3D forms derived from polygons (Carroll, 1993; Oltman, Raskin, Witkin, et al., 1971; Shepard & Metzler, 1971). For example, Shepard & Metzler’s, 1971 seminal work on mental rotation involves the manipulation of 3D figures made up of multiple attached unit cubes. Building on their work, Vandenberg and Kuse (1978) developed a paper-and-pencil test of mental rotation using stimuli similar to those used in the original study. The Mental Rotations Test (Vandenberg & Kuse, 1978) and many other tests measuring individual skills in imagining and manipulating polygon-derived forms, such as the Embedded Figures Test (Oltman et al., 1971) and the Paper Folding Test (Ekstrom, French, Harman, & Derman, 1976), are still commonly used by STEM researchers and educators today (Atit et al., 2013; Held & Hui, 2011).

In contrast to small-scale spatial skills (e.g., mental rotation and paper folding; Ekstrom et al., 1976; Shepard & Metzler, 1971), but of importance in many STEM domains, are large-scale or environmental spatial skills. Environmental spatial skills are involved in everyday tasks, such as finding one’s way in the environment and in learning the layout of a building or a city. Unlike tests of small-scale spatial skills, tests of large-scale spatial skills are generally context-dependent and require solving spatial problems that are situated within the environment (Allen, Kirasic, Dobson, Long, & Beck, 1996; Weisberg & Newcombe, 2016; Weisberg, Schinazi, Newcombe, Shipley, & Epstein, 2014). Typical tasks used to assess these abilities include recognition of scenes from a learned environment, retracing routes taken, sketching a map of the environment, route distance estimates, and pointing to unseen landmarks in the environment.

One kind of large-scale spatial skill that has been commonly examined in studies of STEM teaching and learning is spatial orientation or perspective-taking (e.g., Carbonell Carrera & Saorín Pérez, 2011; Carbonell-Carrera & Hess-Medler, 2017; Kozhevnikov, Hegarty, & Mayer, 2002; Lowrie & Logan, 2018; Tartre, 1990). Perspective-taking is critically important for many STEM domains, such as the earth and space sciences (Hegarty, Crookes, Dara-Abrams, & Shipley, 2010). It involves imagining how a stimulus array will appear from another perspective (Evans, 1980; Lohman, 1988; McGee, 1979). A psychometric test of perspective-taking commonly used in STEM education research is the Object Perspective Test developed by Kozhevnikov and Hegarty (2001). Perspective-taking skills play a role in both field-based (e.g., Tarampi, Atit, Petcovic, Shipley, & Hegarty, 2016) and non-field-based STEM disciplines (e.g., Kozhevnikov et al., 2002; Lowrie, Logan, & Ramful, 2016; Tartre, 1990). Further research is needed to understand how the role of large-scale spatial skills differs between field-based and non-field-based STEM domains.

Only one study, to our knowledge, has examined navigation skills in STEM disciplines. Nazareth, Newcombe, Shipley, Velazquez, and Weisberg (2019) compared the navigational skills of STEM (specifically geologists) and non-STEM experts and examined whether novices’ navigational skills improved after engaging in a spatially demanding STEM task: learning how to use geographic information systems (GISs). Findings of this study revealed that geologists had superior navigation skills to their non-STEM counterparts, and that learning GIS improves students’ navigational skills. Because many of the large-scale spatial tasks are intertwined (e.g., navigation involves the use of spatial orientation skills; Kelly, McNamara, Bodenheimer, Carr, & Rieser, 2008), a question for future research includes deciphering the role of different kinds of large-scale spatial skills in different kinds of STEM-specific spatial problems.

## A shift in the approach to studying spatial thinking

Based upon research on teaching and learning in STEM disciplines, the National Research Council (2006) released a report in 2006 that concluded that spatial thinking was a multifaceted construct. The report argued that while spatial thinking necessarily involves leveraging spatial skills, it is more accurately characterized by analyzing the specific cognitive demands of any given STEM task. The report reframed the discussion to characterize spatial thinking according to the spatial concepts, tools of representation, and processes of reasoning relevant to a specific task. *Spatial concepts*, which are common across the STEM disciplines, refer to the multiple meanings of space (e.g., dimensionality, perspective, distance). *Tools of representation*, which are specific to each discipline*,* refer to the various diagrams, symbols, and software applications used by STEM practitioners to represent spatial concepts and support problem-solving and communication. *Processes of reasoning*, which could involve both common and discipline-specific processes, refer to the cognitive mechanisms by which learners make sense of spatial concepts in different representations to describe and explain a phenomenon or predict how spatial concepts change over time (National Research Council, 2006).

In light of the National Research Council's (2006) framework, many psychological researchers have recognized that spatial thinking, as it had been studied in the past, does not capture the breadth, nuance, or complexity of spatial thinking as it occurs in STEM domains. For instance, regardless of the particular content, STEM learners and practitioners are routinely tasked with mentally simulating rotations of objects or updating their perspective to make predictions (e.g., Hegarty, Keehner, Cohen, Montello, & Lippa, 2007); conversely, these processes of reasoning are often supported by disciplinary representations, such as block diagrams, molecular models, and vector diagrams (e.g., Petcovic, Ormand, & Krantz, 2016). For example, chemists rotate mental images of molecular representations to compare the relative location of two atoms in a structure, and physicists mentally simulate the trajectory of subatomic particles in a particle accelerator using field lines and force diagrams. Although different fundamental spatial skills (e.g., mental rotation, perspective-taking, spatial visualization) are commonly reported by expert scientists (Gilbert, 2005), and their contribution to academic success among STEM learners has been heavily studied (Wai et al., 2009), the interaction between spatial skills assessed by psychometric measures and spatial thinking as carried out by STEM experts remains poorly understood. As a result, there has been a growing interest in the field of psychology in conducting interdisciplinary research on spatial thinking in STEM disciplines. Below, we examine findings from interdisciplinary research in three specific STEM disciplines and reflect on lessons learned regarding the role of spatial skills in these areas of expertise. Simultaneously, discipline-based education researchers have also been trying to ascertain how to bolster students’ skills for completing spatial tasks within their respective disciplines. We will integrate both perspectives to provide a more complete picture of the role of spatial skills in STEM domains.

## Descriptions of STEM expert practice

To demonstrate the rich and varied nature of expert practice in the STEM fields of focus within this paper (i.e., structural geology, surgery, and chemistry), we situated relevant findings within sample scenarios of expert practice within each domain. The sample scenarios of expert structural geology practice discussed here are an aggregate of information from descriptions of and research on expert practice (e.g., Liben & Titus, 2012; Mogk & Goodwin, 2012; Petcovic & Libarkin, 2007; Shipley & Tikoff, 2016) as well as from the first author’s personal experiences and research (e.g., Atit, Gagnier, & Shipley, 2015; Atit, Shipley, & Tikoff, 2014; Tarampi et al., 2016) as an interdisciplinary researcher with a specialty in the geosciences. The sample scenarios of expert practice in surgery were constructed from descriptions of and research on expert and novice surgical practice (e.g., Hegarty et al., 2007; Keehner et al., 2004; Risucci, Geiss, Gellman, Pinard, & Rosser, 2001). Lastly, the sample scenarios of expert chemistry practice are derived from the third author’s personal experiences, expertise, and research (e.g., Stieff et al., 2018; Stieff, Lira, & Scopelitis, 2016; Stieff & Raje, 2010). The third author is a discipline-based education researcher in chemistry and has an educational background in both chemistry and learning sciences. The sample scenarios presented here are not the only scenarios or series of events that could occur during expert practice, but rather were chosen for illustrative purposes on the nature of spatial thinking in each STEM domain.

## Spatial thinking in STEM domains is influenced by domain knowledge and is context-dependent

Unlike the isolated polygon-derived tasks used on most psychometric tests of spatial skills, the spatial problems faced by STEM experts are specific to the realm of their domain and are influenced by the experts’ knowledge of the domain. The expert’s knowledge of the domain influences his approach to solving the spatial problem (Chase & Simon, 1973; Hegarty et al., 2007; Shipley & Tikoff, 2016). In a parallel example to the role of STEM expertise on spatial thinking, the relation between context, prior knowledge, and problem-solving approach has been heavily investigated by psychologists in the field of chess. Chess experts use their prior knowledge of the game to divide and encode the spatial layout of the board as meaningful chunks of information, and then integrate those chunks into an existing mental model of the board from which they generate plausible future moves (Chase & Simon, 1973; Gobet & Simon, 1998).

### The influence of domain knowledge and context on spatial thinking in geology

The substantive spatial problem faced by structural geologists is to use measurements from present-day rock geometries to understand the geologic history of a region. When deducing the geologic history of a region, the structural geologist needs to first identify the geologic structures that exist in the area. They identify the geologic structures by visiting locations where pieces of the structures are visible, known as an outcrop, and collects pertinent data that will inform the geologist about the shape of the geologic form making up the current topography (e.g., Compton, 1985; Mogk & Goodwin, 2012).

The interpretation of topographic maps provides a good example of the interaction between a structural geologist’s prior knowledge and the influences of context on their spatial problem-solving approach. A topographic map contains information about the 3D shape of the topography in a region using contour lines that denote elevation information (Geographic Information Technology Training Alliance, 2016). The structural geologist uses the map to determine whether visible outcrops may be located, to pinpoint their own location in the area, and also to understand how the geologic structure of interest is situated within the region’s topography. When interpreting a geologic map, the expert’s domain knowledge interacts with his or her spatial skills to result in an advanced level of perceptual processing (see Goldstone & Barsalou, 1998 and Barrett, Lindquist, & Gendron, 2007 for further examples on the interaction of perceptual and conceptual processing). For instance, interpreting topographical information on the map involves skill in understanding how elevation information is denoted using contour lines, and also skill in visualizing the represented topography in 3D (Atit, Weisberg, Newcombe, & Shipley, 2016; Liben & Titus, 2012). The expert’s approach to interpreting topographical information involves identifying meaningful patterns in the contour lines that represent topographical structures (e.g., contour lines in concentric circles represent a hill) (Chang, Antes, & Lenzen, 1985), and then visualizing those structures in 3D (Eley, 1981, 1983). Prior experience using topographic maps provides expert geologists with specialized schemas that guide the perception and extraction of 3D information from the 2D representation.

In addition to influencing the extraction of spatial information from a 2D map, the structural geologist’s domain knowledge affects his attention-focusing processes when making sense of large amounts of spatial information. At each outcrop, the expert has to decide which exact location will provide measurements that are informative about the 3D geometry of the geologic structure. Before taking any measurements, the structural geologist first focuses their attention on the critical spatial properties (e.g., the orientation of the rocks on a bedding plane). Focusing attention on the important spatial information involves actively ignoring many other aspects of the scene (e.g., the orientation of the tree on the outcrop or the size of the minerals—a geologic feature not pertinent to the problem at hand). The spatial skill used to identify relevant information for further cognitive processing is called disembedding in the geosciences (Manduca & Kastens, 2012; Reynolds, 2012) and selective attention in psychology (Moran & Desimone, 1985). Understanding which exact spatial properties of an outcrop to attend to is a result of prior knowledge and perceptual expertise (Coyan, Busch, & Reynolds, 2010; Shipley & Tikoff, 2016).

Expert-level knowledge also comes into play in a structural geologist’s memory for spatial locations (Holden, Newcombe, Resnick, & Shipley, 2016). Human memory for spatial locations is often biased. For example, adults recall irregularly shaped spaces or routes as being regular or parallel/perpendicular (e.g., Tversky, 1981) and consistently estimate the distance from point A to point B as different than the distance from point B to point A (e.g., McNamara & Diwadkar, 1997; Newcombe, Huttenlocher, Sandberg, Lie, & Johnson, 1999). Humans also demonstrate biases in their memory for spatial locations—we tend to misremember locations as being more central to the surrounding region than they are (e.g., Holden, Curby, Newcombe, & Shipley, 2010; Huttenlocher, Hedges, & Duncan, 1991). Research in psychology suggests that these errors are due to the tendency to remember locations hierarchically, at both a fine-grained (metric) level and a coarser, categorical level (Huttenlocher et al., 1991). For instance, our memory for the location of our keys is encoded as both the number of inches they are from the edge of the table (metric) and also as their placement on the table in the living room (categorical). For structural geologists, memory for spatial locations at the categorical level is influenced by their prior knowledge. When their memory for locations was tested within images with narrow, geologically defined categories, structural geologists were more accurate in their estimations than organic chemists, who are also experts in a spatially demanding STEM domain. When their memory for locations was tested within images with larger, novice-defined categories, the geologists demonstrated the same amount of error as the other comparison groups (Holden et al., 2016). These findings indicate that expert structural geologists process scenes of geologic interest differently than novices, and use domain-specific information, such as the sedimentary structure of an area, to structure their memory for that location.

Deducing the geologic history of a region requires the structural geologist to visualize the movement of geologic structures that are presently static in space and in time. Visualizing these movements involves understanding that a presently static object may not have always been static. Expert geologists, when asked to interpret diagrams representing bathymetry and topography, i.e., static spatial information, look at the data presented and infer processes that previously took place, such as erosion, faulting, and volcanism. Novices do not (Kastens, Shipley, Boone, & Straccia, 2016). Inferring movement from stationary data requires seeing the relationship between static information and motion (Atit et al., 2014; Kastens et al., 2016; Shipley & Tikoff, 2016). Experts’ understanding of the evolution of geologic structures over time gives them the ability to draw dynamic conclusions (i.e., infer how structures have come to be or will change) from static spatial information, further indicating that prior knowledge shapes STEM experts’ spatial thinking. There is some research that indicates that fundamental spatial skills, such as mental rotation skills, are related to the spatial skills for reasoning about dynamic information presented in static discipline-specific diagrams (e.g., Kastens et al., 2016; Ormand et al., 2014). However, to our knowledge, this research has been conducted only in novices. As prior research indicates that novices struggle to extract dynamic information represented in static formats (e.g., Kali & Orion, 1996; Kastens et al., 2016), research in experts is needed to fully understand the role of fundamental spatial skills in visualizing the movement of information presented in static discipline-specific diagrams.

In sum, the approaches and processes carried out by expert structural geologists when solving spatial problems are influenced by their expertise in the discipline and the contexts in which the problems occur. As demonstrated in the prior examples, for many spatial problems that expert structural geologists try to solve, the roles of domain-specific expertise and context are closely intertwined. Further research is needed to understand the nuances of how domain knowledge interacts with contextual factors to shape experts’ spatial reasoning.

### The influence of domain knowledge and context on spatial thinking in medicine

Interdisciplinary research examining spatial thinking in the field of medicine further demonstrates the interaction between STEM experts’ domain knowledge and the context in which the spatial problems occur to influence experts’ spatial problem-solving. Practicing medicine heavily engages practitioners’ spatial skills (Hegarty et al., 2007; Risucci et al., 2001; Wanzel, Hamstra, Anastakis, Matsumoto, & Cusimano, 2002). Practicing medicine requires a detailed understanding of the 3D spatial properties of anatomical structures and their spatial relations. For instance, medical professionals need to know the 3D shape of the structures (e.g., the shape of the kidney), their locations relative to each other (e.g., the location of the kidney relative to the bladder), and how they are connected (e.g., the location and path of the ureter, the tube that connects the kidney to the bladder). Similar to geologic structures, internal anatomy is usually not directly visible. Furthermore, the exact properties of the structures, such as the shape and location, vary from person to person (Hegarty et al., 2007). Therefore, when carrying out a medical procedure, the medical professional heavily relies on his or her prior knowledge of the human body.

Within the field of medicine, conducting surgery is an example of a complex spatial problem. Surgeons have been recognized for their skill in completing intricate spatial tasks (Gibbons, Baker, & Skinner, 1986; Risucci, 2002). They use their spatial skills, coupled with fine motor skills, perceptual expertise, and domain knowledge, to navigate small areas within the body using surgical tools during operations. The task of navigating the human body during operations becomes even more difficult in surgeries that are minimally invasive (Hegarty et al., 2007), such as during laparoscopic surgery.

Laparoscopic surgery is conducted through small incisions on the body that may be far from the target organ site. Generally, this surgery is conducted in the abdominal or pelvic cavity. It is the procedure of choice for many doctors and patients due to its clinical benefits, such as its short recovery time, and decreased pain and hemorrhaging. Though beneficial for the patient, conducting this surgery is known to be challenging for the surgeon (Tendick et al., 2000). During laparoscopic surgery, the surgeon makes several small incisions in the patient’s abdominal wall, each large enough to accommodate a narrow tube or cannula. Through one of these incisions, a laparoscope, a camera mounted on a long tube with internal optics, is passed into the abdominal cavity. Long-handled surgical instruments are then inserted through the other incisions. The laparoscope transmits a video image from inside the abdomen to a monitor, which the surgeon uses to guide activities and maneuver instruments at the target site (Hegarty et al., 2007).

Accurately using the information presented on the visual display involves coordinating information from multiple different reference frames. The visual information presented on the display is placed at a distance from the location of the operation on the body. Thus, the surgeon has to map the visible features on the screen to the spatial framework of the structures in the body, and then to the spatial framework of his own mental model of the relevant anatomy (Loomis, Klatzky, & Lederman, 1991; Wu, Klatzky, & Stetten, 2010). Coordinating across multiple reference frames is a source of errors in judgments of depth and in estimating the 3D geometry of internal objects (e.g., organs inside the body) (Wu et al., 2010). Hence, the surgeon’s prior knowledge allows for a higher level of cognitive processing that keeps the surgeon from making these spatial errors during surgery. However, the details of how a surgeon’s domain knowledge alters her cognitive processing to decrease the occurrence of spatial errors are not well understood. Further research on the interaction between surgical experts’ domain knowledge, cognitive processes, and the context of the spatial problem is needed to ascertain the role of surgical expertise on spatial problem-solving in surgery.

### The influence of domain knowledge and context on spatial thinking in chemistry

Unlike the examples of structural geology and laparoscopic surgery, where the context in which the spatial problems occur is the visible environment, such as the human body on the surgical table, many spatial problems in STEM occur in contexts that are invisible to the problem-solver. Chemists solve spatial problems pertaining to the study of the composition, structure, properties, and change of matter. Studying matter at this level involves reasoning about miniscule abstract 3D phenomena, such as atoms and molecules, which are not visible or tangible to the problem-solver. Furthermore, these phenomena behave in ways that are fundamentally different from those of physical objects on larger scales (Johnstone, 1982, 1991). To understand and manipulate objects at the submicroscopic level, experts in the discipline heavily rely on the use of 2D representations to scaffold their thinking and support mental simulations of chemical events (Keig & Rubba, 1993; Stieff, 2004).

In chemistry, 2D domain-specific symbols, figures, formulae, and diagrams are commonly used to represent 3D chemical entities, such as atoms and molecules, and their associated phenomena (e.g., Cheng & Gilbert, 2009; Gilbert & Treagust, 2009; Goodwin, 2008; Kozma, Chin, Russell, & Marx, 2000; Treagust & Chittleborough, 2001). These representations are ubiquitous across the discipline. “Chemists use them to design research protocols, interpret experimental results, and communicate their ideas” (Stieff, 2004, p. 13). Successful reasoning in the domain, including solving context-specific spatial problems, involves knowing how to decode the 3D information embedded in these representations, and also knowing how to translate information between multiple different representations (Stieff, 2004; Stieff & Raje, 2010; Stull, Hegarty, Dixon, & Stieff, 2012).

The use of 2D representations in solving complex field-relevant spatial problems is particularly important and common in organic chemistry (Habraken, 1996; Stieff, 2007). Organic chemistry is the study of the structure, properties, and reactions of organic compounds and organic materials (i.e., matter in its various forms containing carbon atoms). This subdiscipline has been the focus of research on spatial thinking in chemistry because it is in the organic chemistry course in college that instructors begin to strongly emphasize the relationship between 3D molecular structures and chemical reactivity repeatedly throughout the curriculum (Stieff, 2004).

Solving spatial problems in organic chemistry entails knowledge and skill in using the different kinds of 2D representations to predict 3D structures (Gilbert & Treagust, 2009; Wu & Shah, 2004). This is particularly salient in practices related to organic synthesis. The work of a synthetic organic chemist involves planning a reaction scheme that will produce a target molecule with specific 3D structural features from a series of chemical reactions and related purifications. The chemist often begins work with common disciplinary representations (e.g., structural diagrams, Fischer projections, and chair representations) to create a plan that typically involves combining, transforming, and decoupling several unique small molecules to generate a larger, more spatially complex molecule. The most common strategy for generating this plan is to develop a “retrosynthetic scheme” (Corey & Cheng, 1996) that involves working backwards from the target molecule by cleaving it into ever smaller molecules until the chemist identifies starting materials that are commercially available for use. This process can generate multiple, competing schemes. From here the chemist starts forward planning by proposing a series of chemical reactions that will gradually convert the starting materials in sequence to the desired target structure.

In sum, examining spatial thinking in structural geology, surgery, and chemistry underscores how the spatial problems faced by STEM experts are derived from the contexts in which they occur. It also highlights that the approach and processes STEM experts carry out to solve spatial problems are influenced by their prior knowledge and expertise in the domain. What is unknown and needs to be answered by future research is the extent to which fundamental spatial skills, those generally assessed by psychometric measures, are needed to support context-dependent spatial thinking at advanced levels of STEM reasoning. Furthermore, longitudinal research is necessary to understand how spatial skills evolve as a student progresses from STEM novice to expert. Answers to these questions would inform the development of interventions and supports for STEM learning and practice.

## Fundamental spatial problems increase in complexity when they occur within STEM contexts

Fundamental spatial skills—those that are the focus of psychometric measures of spatial thinking—include skills such as spatial perception and mental rotation. STEM experts, when asked to reflect on the cognitive processes conducted to solve discipline-specific problems, often describe engaging these fundamental spatial skills (Gilbert, 2005) but with an increased level of complexity and nuance than presented in the original paper and pencil tasks.

### Complexity of spatial problems in structural geology

For instance, in tests of spatial perception, participants are required to determine spatial relationships with respect to the orientation of their own bodies, in spite of distracting information (Linn & Petersen, 1985). Psychometric measures assessing this skill include the Water Level Task (Liben, 1991), which requires participants to draw a horizontal line in a tilted bottle, and the Rod and Frame Test (Witkin, Dyk, Fattuson, Goodenough, & Karp, 1962), in which participants must place a rod vertically while viewing a frame oriented at 22 degrees. Although the original descriptions of this spatial skill implied universal understanding of horizontality by adolescence (Piaget & Inhelder, 1956), many individuals continue to find spatial perception problems difficult even into adulthood (Rebelsky, 1964; Thomas, Jamison, & Hummel, 1973). Structural geologists use spatial perception skills when interpreting a Brunton compass. A Brunton compass is a precision instrument that is used to make degree and angle measurements in the field. The geologist has to take two kinds of measurements using the compass: the strike of the rock and the dip of the rock. The strike is the horizontal direction of the layers of rock, and the dip is the direction in which the rocks are tilted (Compton, 1985; National Park Service, 2019). Taking accurate strike and dip measurements at an outcrop involves having a strong conceptual understanding of two fundamental spatial concepts: horizontal and vertical (Liben & Titus, 2012). Strike and dip are both measurements of the amount of deviation from those planes. These measurements provide critical data about the 3D geometry of the rocks, which inform the geologist about their deformational history (Compton, 1985; Liben, Kastens, & Christensen, 2011). Structural geology novices report that strike and dip are among the most troublesome concepts in introductory geology (Hemler & Repine, 2006).

We speculate that there are a number of factors that make measuring strike and dip in the field a more complex spatial task than that required by the Water Level Task. First, in addition to using spatial perception skills, taking accurate strike and dip measurements involves the simultaneous engagement of other spatial skills, such as disembedding skills. Disembedding skills are necessary to identify specific locations within a larger area of noise where measurements would provide the most information about the 3D geometry of the larger geological structure (see discussion on disembedding in the section titled "The Influence of Domain Knowledge and Context on Spatial Thinking in Geology"). Additionally, unlike the Water Level Task and the Rod and Frame Test, strike and dip measurements are taken on uneven surfaces potentially covered with plants, grass, or other vegetation which is naturally occurring in the environment. These materials on the potentially jagged surface of the outcrop make taking measurements and extracting planar information a more difficult task than that required by psychometric tests of spatial perception. In the psychometric tests of spatial perception, the context in which judgments of horizontality and verticality are made is uniformly tilted. No studies, to our knowledge, have directly investigated why measuring the strike and dip of an outcrop in the field is such a challenging task, especially for novices (e.g., Hemler & Repine, 2006; Ishikawa & Kastens, 2005). A cognitive task analysis involving both experts and novices when doing fieldwork would shed light on the various components that are involved in taking these fundamental measurements in the field.

### Complexity of spatial problems in medicine

Mental rotation is a spatial skill involving the rotation of 2D and 3D objects rapidly and accurately. It is commonly measured using psychometric measures such as the Mental Rotations Test (Vandenberg & Kuse, 1978) and the Purdue Spatial Visualization Test: Rotations (PSVT:R; Guay, 1977). In the Mental Rotations Test participants are asked to identify two figures that are rotated versions of the target figure (Vandenberg & Kuse, 1978). In the PSVT:R, participants view two images of an object, one in its original position and one after it is rotated, and are asked to select the correct pairing of an analogous set of objects rotated in the same manner as the first set. Surgeons engage in mental rotation when making sense of the information presented in surgical displays. The image of the human anatomy presented on the screen is in a different perspective from that of the surgeon (Hegarty et al., 2007). During the procedure, the surgeon moves the laparoscope to get more information about the patient’s anatomy, or places it on an angle that provides the necessary view. The images from the movements of the camera or the angle in which it is positioned show a different perspective of the anatomy than the perspective of the surgeon’s mental image. Therefore, to accurately integrate the information on the screen into her mental picture, the surgeon needs to mentally rotate the image and her mental model until the perspectives align.

Aligning the two perspectives is even more spatially challenging if the surgeon used a laparoscope with an angled lens. Using a laparoscope with a straight objective lens limits the area visible to the expert to the region located right in front of the lens. Insertion through the abdominal wall creates a fulcrum that limits the laparoscope’s range of possible motions. So, a straight lens provides an extremely restricted range of viewing perspectives. For many procedures, this limitation is overcome with an objective lens fitted at an angle (e.g., 45 degrees) with respect to the laparoscope’s longitudinal axis. This expands the field of view considerably and allows the surgeon to look from underneath, above, and partly around internal structures as the scope is rotated (Hegarty et al., 2007). Although it allows the surgeon to view the anatomy from a number of different perspectives, it also adds complexity to the already spatially demanding task of aligning the picture on the screen to the perspective of the surgeon’s mental image. Now, the expert has to account for the deviation from the angled lens when aligning the perspective of the picture to that of his mental image. Though consisting of more complex spatial transformations than those measured in psychometric assessments of spatial skills, researchers have found that skill in using an angled laparoscope in novices is correlated with their performance on measures of mental rotation and paper folding (Eyal & Tendick, 2001). How to support surgical students who have weaker fundamental spatial skills, and determining whether training fundamental spatial skills improves their laparoscopic surgery performance are questions for future research.

### Complexity of spatial problems in chemistry

In the field of chemistry, though many chemists use analytic or algorithmic strategies for familiar molecular structures, mental rotation is a key cognitive process used to make identity judgments and compare novel molecular structures (Stieff, 2007). Such identity judgments typically involve examining disciplinary representations to compare the absolute configuration (i.e., the relative spatial location of atoms within a molecule) of two molecules to establish whether the two molecules are identical, constitutional isomers (i.e., molecules made of the same constituent atoms that differ in connectivity), or stereoisomers (i.e., molecules made of the same constituent atoms with identical connectivity that differ in the spatial relationships among those atoms). Such judgments require explicitly reasoning about spatial information to perform imagined spatial operations, such as mental rotation, to make an identity judgment. Factors such as the axis of rotation of the pertinent molecules and the spatial complexity of the molecules to be rotated influence task difficulty (Stieff et al., 2018).

While early instruction in chemistry involves problems that tap fundamental skills related to mental rotation (e.g., Habraken, 1996), the complexity of such problems increases significantly and rapidly in the curriculum. Students must learn to visualize how molecules undergo dynamic spatial transformations that allow them to fold and spin in a variety of ways that produce complex spatial conformations. In turn, these conformations can alter the energy of the structure and subsequent reaction pathways. Complex synthesis problems require students to anticipate how the 3D structure of a molecule can prohibit or facilitate binding with other molecules to produce a desired outcome or to prevent unwanted side reactions. This requires a complicated interplay between visualization, perspective-taking, mental rotation, and representational competence for successful problem-solving.

Even though fundamental spatial skills are used by STEM experts in solving field-relevant spatial problems, studies have found that bolstering students’ fundamental spatial skills does not result in improving their skills for carrying out their domain-specific applications (Stieff, Dixon, Ryu, Kumi, & Hegarty, 2014; Stull et al., 2012). Research examining the tools and strategies that mediate the relationship between fundamental spatial skills and their domain-specific applications is necessary to understand how to develop the advanced levels and complex forms of fundamental spatial skills in future STEM practitioners.

## STEM practice involves spatial skills distinct from those captured by existing psychometric measures

Research on fundamental spatial skills has relied heavily on a few measures (e.g., the Mental Rotations Test) which sample only a few of the many types of spatial problems STEM practitioners face regularly in their occupations. More recent findings from interdisciplinary research have revealed that STEM practitioners are often faced with spatial problems that involve processes that the field of psychology has not yet defined. Much of this research has been in the field of structural geology, where the shapes and spatial properties of the geologic structures found in the environment dictate the kinds of spatial problems a structural geologist has to solve.

When measuring the orientation of the rocks at an outcrop, a structural geologist has to integrate data from multiple faces of the structure to infer its 3D geometry under the ground. Predicting the location of invisible individual elements of an object using visible data points is referred to in the geosciences as penetrative thinking (Alles & Riggs, 2011; Kali & Orion, 1996). Novices to the geosciences find penetrative thinking difficult as they struggle to combine spatial information from multiple faces of an object. They often mistake the spatial properties they see on one face as the properties that extend straight through the rest of the structure (Gagnier & Shipley, 2016). On the other hand, experts override the instinct to infer the internal geometry from only one face, and instead incorporate multiple pieces of visual and spatial data to make their predictions. When trying to understand what kinds of spatial skills are involved in penetrative thinking, researchers have found that psychometrically assessed spatial skills, such as mental rotation and perspective-taking, are not predictive of penetrative thinking skills (Gagnier & Shipley, 2016). Thus, the spatial skills involved in penetrative thinking are distinct from the fundamental spatial skills that are believed to underlie STEM learning (Uttal & Cohen, 2012; Wai et al., 2009), and perhaps are developed with extensive experience inferring the geometry of largely unobservable geologic structures. As surgeons also have to estimate the 3D geometry of internal organs (Hegarty et al., 2007), a question for future research is whether the corpus of spatial skills pertinent to surgery also includes penetrative thinking. An answer to this question has potential broad implications regarding the kinds of spatial skills to be developed in students during early education.

The spatial skills used by structural geologists when conducting non-rigid mental transformations are also unlike the polygon-derived spatial skills that are commonly studied by psychologists. To understand the geologic history of a region, the structural geologist visualizes the transformations the region underwent to result in the present topography. For instance, if the region has a geologic fold (a U-shaped geologic structure), then the structural geologist has to visualize the geologic events that occurred to cause the once-flat layers of rock to fold upward. Visualizing the events that caused the layers to fold involves imagining the bending or unbending of an object. Bending and unbending involve a non-rigid mental transformation, a transformation in which the distance between two points on the object changes. Psychometric measures of spatial thinking commonly employ tasks involving rigid mental transformations (Hegarty & Waller, 2004; Shepard & Metzler, 1971), transformations in which the distance between two points on an object stays the same, such as in mental rotation. Work by Atit et al. (2013) as well as Resnick and Shipley (2013) shows that skills for rigid and non-rigid mental transformations are separable, indicating that the current psychometric measures of spatial thinking are not capturing all of the spatial skills earth scientists use to solve the spatial problems they encounter within their domains.

A key idea here is that the STEM domains can provide the requisite categories of spatial skills, and psychologists are benefiting from looking to STEM domains to understand the kinds of spatial problems that exist in the natural world. Further research involving collaborations between STEM and psychology are needed to further our understanding of the nature of spatial thinking in STEM domains. Moreover, since spatial skills have been found to predict success in STEM domains (Shea et al., 2001; Wai et al., 2009), identifying the kinds of problems STEM experts solve during their practice and categorizing them could guide new approaches to curriculum development aimed at bolstering students’ spatial skills to foster STEM learning.

## Sometimes experts do not rely on spatial skills to complete spatial tasks

Much of the research in psychology indicating that spatial skills are critical for STEM learning and practice makes a strong case for the importance of spatial skills in predicting who goes into STEM fields and who stays in STEM (Shea et al., 2001; Wai et al., 2009). However, at the expert level of STEM practice, many problems that appear to be spatial in nature or may require the engagement of spatial skills by a novice are solved with little or no use of spatial skills by experts (Hambrick et al., 2012; Stieff, 2007; Uttal & Cohen, 2012). Hambrick et al. (2012) investigated the relations between experts’ and novices’ psychometrically assessed spatial skills and their performance on a field-relevant geoscience task: bedrock mapping. Participants’ expertise, or geologic knowledge, was measured using performance on a mapping questionnaire and a geologic knowledge questionnaire. In the bedrock mapping task, both expert and novice geologists were asked to map out the underlying structures in a given area, based on observable surface features, on a blank map. Spatial skills positively predicted bedrock mapping performance at low, but not at high levels of geological knowledge (Hambrick et al., 2012).

Similar findings are reported in the fields of laparoscopic surgery and in chemistry. Keehner et al. (2004) compared performance on a test of paper-folding, videoscopic experience, and operative skills between students enrolled in an advanced course for experienced surgeons and novice students enrolled in a laparoscopic urology surgery course. Results revealed that paper-folding performance was correlated with operative skills in novices but was not related with operative skills in experienced surgeons (Keehner et al., 2004). Stieff (2004, 2007) investigated expert and novice chemists’ performance on a mental rotation task consisting of both the classic Shepard and Metzler (1971) 3D block figures and representations of 3D chemical molecules. Novice and expert chemists performed nearly identically on the Shepard and Metzler figures. In both groups, there was a strong linear relation between the degree of angular disparity and reaction time—a result often taken as evidence of mental rotation. However, there was a strong expert-novice difference for the representations of 3D symmetric chemical molecules. The novices again showed the same relation between angular disparity and reaction time, whereas in experts, this relation was flat (the correlation was nearly zero) (Stieff, 2004, 2007). While novices solve 3D molecular structures using brute mental imagery (Stieff, 2011), the experts’ familiarity with the disciplinary representations and molecular structures allows them to use algorithmic or analytical strategies.

These results suggest that individuals with high amounts of domain knowledge (i.e., experts) may rely on spatial skills less when completing domain-specific spatial tasks or that experts’ spatial skills are attenuated as they interact with spatial problem-solving strategies unique to their disciplines. Interdisciplinary research and DBER examining the role of spatial skills across various spatial tasks within a discipline could help identify discipline-specific tasks where spatial thinking is critical versus tasks that can be more efficiently solved using other strategies.

## Implications for STEM Education

The interdisciplinary research and DBER discussed here shed light on the context-dependent and domain knowledge-integrated nature of spatial thinking in STEM disciplines. Furthermore, this research highlights that, in many instances, the spatial problems STEM experts solve as part of their practice are unlike the spatial problems in psychometric measures assessing spatial thinking skills used by researchers. The discrepancies between the skills required for practice and the fundamental spatial skills defined and categorized by the scientific fields that study them (e.g., psychology) pose a critical question for consideration with regard to preparing the next generation of STEM professionals: What is the best way of developing the spatial skills that students need for STEM success? The findings from studies examining spatial thinking in the fields of structural geology, surgery, and chemistry have led us to the following conclusions.

First, more researchers studying the connections between spatial thinking and STEM domains should ground their investigations within STEM practices to understand the range of spatial skills required in those domains. Tests of fundamental spatial skills have largely been developed outside of STEM domains (Guay, 1977; Shepard & Metzler, 1971) and thus do not capture the variety of skills utilized by practitioners when working in their disciplines (Atit et al., 2013; Resnick & Shipley, 2013). Moreover, the psychometric measures do not account for the context-dependent nature of spatial thinking in STEM. They are devoid of the domain-specific attributes of the problem and thus do not include the complexity resulting from the domain-specific context in which they occur. Though some psychologists have pursued interdisciplinary partnerships with DBER experts and have used a bottom-up approach to studying spatial skills (i.e., examining the kinds of spatial problems that occur within STEM disciplines and the spatial thinking carried out by experts to solve them; Atit et al., 2013; Shipley & Tikoff, 2016; Stieff & Raje, 2010), it is still the uncommon approach to studying spatial thinking in STEM. Many more studies grounded within STEM domains are needed to truly understand the kinds of fundamental and discipline-specific spatial skills students need for STEM success.

Second, in addition to training students’ fundamental spatial skills with the aim of improving STEM outcomes (Cheng & Mix, 2014; Sorby, 2007), students’ field-specific spatial skills need to be trained to facilitate advanced levels of STEM reasoning and understanding. Due to the context-dependent nature of spatial thinking in STEM, it is unclear whether the discipline-specific spatial skills required for practicing one STEM domain would generalize to practicing another STEM domain (e.g., are the penetrative thinking skills used in geology related to the spatial skills used to estimate the location and shape of internal organs used in surgery?). Furthermore, studies have shown that general spatial training in students improves their performance in STEM disciplines (Cheng & Mix, 2014; Miller & Halpern, 2013; Piburn et al., 2002; Sorby, 2007; Sorby, Veurink, & Streiner, 2018), but these outcomes have been largely limited to grade school or introductory levels of STEM learning. At more advanced levels, Ormand et al. (2017) examined whether training both fundamental and domain-specific spatial skills through their Spatial Thinking Workbook boosts spatial thinking in geology majors for three different undergraduate courses: mineralogy, structural geology, and sedimentology. Results showed that completing the workbook exercises improved students’ fundamental spatial skills over the time of the course. Additionally, their data suggested that students who completed the workbook were able to solve domain-specific spatial tasks. However, since no comparisons were made to a control group in their 2017 publication, no conclusions on the effectiveness of their training on advanced-level geological reasoning can be made.

Thus, to our knowledge, no studies have found that training students’ fundamental spatial skills improves STEM learning and performance at more advanced levels (i.e., at the upper-level undergraduate and graduate levels), and prior research has shown that reliance on fundamental spatial skills decreases as more domain-specific knowledge is acquired (Hambrick et al., 2012; Keehner et al., 2004). The lack of evidence showing transfer between fundamental spatial skills and advanced levels of STEM reasoning may be due to the fact that far transfer is difficult to achieve (Stieff & Uttal, 2015), evidenced by the numerous studies attempting to improve intelligence by training working memory skills (Melby-Lervåg, Redick, & Hulme, 2016; Redick et al., 2013). Perhaps increasing the focus of research on spatial thinking to include understanding how to train and improve field-relevant spatial skills in students, as attempted by Ormand et al. (2017), may shed light on how to achieve greater improvements in student STEM outcomes than shown by studies training fundamental spatial skills.

The National Research Council (2012a, b) *Framework for K-12 Science Education* offers some guidance on how training students’ spatial skills to support STEM learning at all levels might be accomplished. Based upon research on teaching and learning in STEM disciplines, the NRC concludes that K-12 science and engineering education should be built around three major dimensions: (1) scientific and engineering practices, (2) crosscutting concepts, which are commonalities in concepts and skill sets across the various science and engineering fields, and (3) core disciplinary ideas, which are concepts and skill sets specific to each of the science and engineering domains (National Research Council, 2012a). We believe that spatial thinking as pertinent to STEM domains follows this framework.

As described above, fundamental spatial skills are processes of reasoning that span STEM disciplines. As such, they mirror the scope of crosscutting concepts critical to reasoning and understanding across the STEM domains. For instance, mental rotation is critical to problem-solving in chemistry (e.g., Harle & Towns, 2011; Stieff, 2007) and surgery (Hegarty et al., 2007), as well as mathematics (e.g., Gilligan, Hodgkiss, Thomas, & Farran, 2017; Lombardi, Casey, Pezaris, Shadmehr, & Jong, 2019; Lowrie & Logan, 2018). On the other hand, field-relevant spatial skills that involve specific tools of representation reflect disciplinary core ideas that are important for and specific to reasoning and understanding within the STEM discipline of interest, such as skills for interpreting 2D diagrams in chemistry (e.g., Padalkar & Hegarty, 2015; Stieff, 2011; Stull & Hegarty, 2016) and skills for interpreting topographic maps in geology (e.g., Atit et al., 2016; Chang et al., 1985; Eley, 1983). As outlined by the National Research Council (2012a) framework, both crosscutting concepts (e.g., fundamental spatial skills) and core disciplinary ideas (e.g., field-relevant spatial skills) are needed for meaningful learning within the STEM disciplines. Accordingly, new learning environments could be designed that emphasize fundamental spatial skills in classrooms at the grade school and introductory levels. This focus would promote the development of crosscutting spatial skills critical for gaining fundamental STEM knowledge before specialized instruction. Later, field-relevant spatial skills should be emphasized in advanced-level STEM classrooms to facilitate the development of disciplinary spatial skills important for expert-level disciplinary knowledge and practice. For instance, domain-specific spatial problem-solving is a topic of emphasis in many upper division earth science courses. In sedimentology and stratigraphy courses, students learn to visualize 2D slices of complex 3D objects, such as fossils and conglomerates (e.g., Hickson, 2005). In structural geology courses, students learn to visualize the different movements of geologic structures, such as fault slips and fault separation (e.g., Tewksbury, 2019). Moreover, many of these courses include the completion of relevant fieldwork experience which provides students with experience in completing spatial tasks within the complex environments in which they naturally occur (e.g., Kastens et al., 2009; Petcovic, Stokes, & Caulkins, 2014). Focusing on the development of upper-level students’ spatial thinking within the context of the domain they are learning (e.g., through embedded spatial exercises) would provide the students with both the spatial skills and domain knowledge required for further advancement in the field (see Piburn et al., 2002 for an example).

Lastly, performance on psychometric tests of spatial skills may not be the only reliable predictor of success in STEM domains. As discussed previously, the limited focus of these tests on general processes of reasoning occludes the complexity of spatial thinking as it occurs in STEM disciplines. Students’ skills for reasoning about field-specific spatial problems should also be taken into consideration. Because spatial thinking at the expert level in STEM domains is influenced by the expert’s domain knowledge and is context-dependent, students who find completing fundamental spatial tasks difficult may still succeed in STEM with the appropriate scaffolds and supports. For example, the use of ball and stick models in organic chemistry facilitates student performance on diagram translation problems (Padalkar & Hegarty, 2015), and interactive animations in structural geology bolsters student understanding of 3D geologic structures represented in topographic maps (Reynolds et al., 2005). Thus, future research should focus on identifying discipline-specific spatial reasoning measures, as well as scaffolds and tools that support students’ spatial problem-solving for each STEM field.

## Conclusion

Spatial thinking is a critical component of STEM learning and practice. Much of what we have learned about this skill set through psychological research only provides a snapshot into the kinds of skills students need to succeed in STEM domains. Close examination of the kinds of spatial problems STEM experts need to solve, the approaches they take to solve them, and how they align with the kinds of spatial problems conventionally studied by psychologists highlights the need to continue broadening the scope of how spatial thinking is commonly studied. Additionally, it sheds light on the benefits of working with disciplinary education experts and building collaborations between psychology and STEM faculty to fully understand the nature of spatial thinking in STEM and to identify the kinds of spatial scaffolds and supports students need to become experts in each field.

## Data Availability

Not applicable.
